# Perioperative observations of different bypass modes of a right coronary system based on instantaneous blood flow during the operation

**DOI:** 10.1186/s13019-020-01229-5

**Published:** 2020-08-14

**Authors:** Zhou Zhao, Chun Fu, Li-xue Zhang, Guo-dong Zhang, Yu Chen

**Affiliations:** 1 Cardiac Surgery Department, Peking University People’s Hospital, Beijing, 100044 China; 2 Critical Care Medicine Department, Peking University People’s Hospital, Beijing, 100044 China

**Keywords:** Coronary artery bypass grafting, Transit-time flowmeter, Sequential grafts

## Abstract

**Background:**

With the ageing of China’s population, the incidence and mortality of coronary atherosclerotic heart disease (CAD) is increasing year by year, which brings a heavy burden to the family and society [1]. We aimed to analyse the strategy of coronary artery bypass grafting (CABG) in the right coronary artery and to compare the haemodynamic characteristics of the sequential grafts with those of single grafts and to observe the patency rate of those grafts for 1 week after the operation.

**Methods:**

A total of 242 patients (178 men, mean age 62.6 ± 8.8 years) underwent right coronary artery bypass grafting in our hospital from October 2016 to January 2019. The blood flow (Q, ml/min), pulsatility index (PI) and related parameters of the grafts were measured and recorded by TTFM during the CABG. The patency of the grafts was evaluated by coronary computed tomography (CT) for 1 week after the operation.

**Results:**

The most common material used for the graft in the right coronary system of CABG is the greater saphenous vein (92.3%), followed by the radial artery (5.5%) and the internal mammary artery (1.9%). The highest frequency target of the right coronary artery is the posterior descending artery (PDA) (47.6%), followed by the right main coronary artery (RCA) (29.1%) and the posterior branch of the left ventricle (PL) (23.3%). The proportion of single grafts was the highest for the right coronary artery in CABG (178 cases, 67.9%), followed by a graft of the PDA-PL (42 cases, 16.0%) and other sequential grafts among the different coronary artery systems (including the system of the left anterior descending artery (LAD) and the left circumflex (LCX)). Whether there were sequential grafts of the PDA-PL or other sequential grafts among the different systems of the coronary artery, the instantaneous flow of a group of sequential grafts was higher than that of a single graft, and the difference had statistical significance (*P* < 0.01). However, there were no significant differences in the flow between the groups of sequential grafts (*P* = 0.410). Diastolic flow (DF) in the group of sequential grafts of the right coronary system was better than that in the non-sequential group (*P* < 0.001), and the difference had statistical significance. There was no significant difference between the DF of the groups of the other system of sequential grafts and that of the right coronary sequential grafts. Coronary artery CT suggested that there were 11 cases of poorly developing grafts or stenosis and occlusion a week after the operation, and those phenomenon mainly occurred in the group with a single graft. There was only one case that was occluded in the group of other systems of sequential grafts, and statistically significant differences existed between the two groups (*P* < 0.01).

**Conclusions:**

In our centre, the most common form of CABG in the right coronary artery system is a non-sequential vein bridge to the PDA. Whether there are sequential grafts of the PDA-PL or other sequential grafts among the different coronary artery systems, the instantaneous flow of a group of sequential grafts is higher than that of a single graft. DF in the group of sequential grafts of the right coronary system was better than that in the non-sequential group.

## Background

With the ageing of China’s population, the incidence and mortality of CAD are increasing year by year, which brings a heavy burden to the family and society [1]. CABG has been recognized as one of the accepted standard protocols for the treatment of complex, multivessel coronary vasculopathy since it was first introduced in the 1970s [2, 3].

The surgical strategies for the right coronary system (including the right main trunk, the posterior descending branch, and the left ventricle branch) have been significantly individualized and differentiated [4], especially in the selection of target vessel sites. The strategy depends on the surgeon’s clinical experience and habits because there is no recognized consensus model for the bypass or surgical strategy of the right coronary system. The purpose of this study was to observe and analyse the surgical strategy of the right coronary artery bypass graft in the conventional CABG population in our centre. The haemodynamic characteristics of the sequential bypass and non-sequential bypass vessels were compared and the patency rate at 1 week after the operation was observed.

## Research materials and methods

### Research data

From October 2016 to March 2019, a total of 242 patients underwent coronary artery bypass surgery in our hospital, including 178 men and 64 women, with an average age of 62.6 ± 8.8 years. The average bypass count was 3.22 ± 0.80. A transient-flow flowmeter (TTFM) was used in all selected patients to measure the vascular flow parameters, and the corresponding results were recorded. Patients who used the radial artery as a bridge vessel were given nicardipine intravenously during the perioperative period, and they started taking diltiazem hydrochloride tablets orally (30 mg three times/day) after removal from mechanical ventilation.

A coronary CT examination was performed 1 week after the operation to observe the patency of the right coronary artery. All patients were routinely taking aspirin 100 mg/day to the day before surgery, and routinely taking aspirin 100 mg/day and plavix 75 mg/day after removal from mechanical ventilation.

### Methods

#### Research methods

TTFM was used to measure and record the related parameters, such as the mean graft flow (MGF) and pulsatility index (PI value). A coronary CT examination was performed 1 week after surgery. Inclusion criteria: 1, simple CABG patients; 2, the right coronary system for coronary artery bypass grafting; exclusion criteria: 1, patients with minimally invasive small incision single and/or multiple coronary artery bypass grafts; 2, patients, for various reasons, who did not undergo TTFM examinations during the operation; and 3, patients who, 1 week after surgery, did not undergo coronary CT examinations for various reasons. All CABG operations were completed by three surgeons with seniority greater than the deputy chief physician, and their independent completion of CABG surgery exceeded 700 cases.

#### Surgical methods

All patients underwent endotracheal intubation combined with general anaesthesia. All patients underwent routine mid-opening. Low-frequency electrosurgical detachment of the left (right) lateral mammary artery and/or brachial artery was conducted under endoscope or direct vision. The saphenous vein collection and the coronary artery bypass grafting occurred under cardio-hepatic cardiopulmonary bypass or non-stop jumping.

#### Instruments and methods

The transient blood flow meter (TTFM) used during the surgery is the Medidist VQ2011 model. After the anastomosis of all of the bypass vessels was completed, the protamine was neutralized and stabilized according to the diameter of the bypass. The ultrasonic probe was selected according to the diameter of the bypass, and the bypass vessel was placed in the probe near the anastomosis for direct measurement [5]. Parameters obtained during surgery: 1. MGF is the average blood flow per minute in the graft bypass blood vessels, and the unit is ml/min. Some studies have suggested that MGF < 15 ml/min is one of the predictors of a poor short-term prognosis [6]. 2. PI is the ratio of the maximum blood flow and the minimum blood flow difference to the mean flow rate [PI = (Q max −Q min)/Qm] in the graft vessel. It is one of the most commonly used indicators of graft function and is used to evaluate CABG with intraoperative TTFM. Excessive PI values represent functional defects in the transplanted blood vessels [7]. Different studies have different definitions of cut-off values for PI. It is generally considered that greater than 5 is considered to be an independent risk factor for graft vascular dysfunction [6]. 3. DF is the ratio of the diastolic blood flow to the sum of the systolic and diastolic blood flow [DF = Q diastole/(Q systole+Q diastole)], which is a good indicator for evaluating the function of the CABG graft. It is generally believed that DF > 50% is one of the indicators of good graft function [8].

#### Intraoperative determination of flow satisfaction criteria

1. The coupling degree is satisfactory when measuring the graft bypass vessel (> 50% or more); 2. The TTFM shows that the blood flow waveform is stable and reproducible; 3. The average flow red line is recorded after the plateau period is stable; 4. According to a previous study, display PI value < 5, flow rate > 15 ml/min is a satisfactory bypass blood flow parameter.

#### Postoperative coronary CT evaluation method

The vascular patency or restenosis was evaluated by two senior radiologists. The diameter of the bypass was measured in the cross-section of the bypass vessel. The mean value of the vascular diameter of the stenosis was not used as a reference. According to the Fitzgibbon bypass vascular grading criteria [9]: Grade A, bypass vessel no stenosis or stenosis < 50%; grade B, vascular stenosis ≥50%, but not completely occluded; grade O, complete occlusion of the bypass vessel. In this study, we classified the grade A into the patency group, classified the grade B and grade O into the stenosis group, and classified the poorly developed ones into the stenosis group.

#### Statistical analysis

All recovered data were analysed using EpiDate3.1 software, and the data were entered in parallel twice. The input data were logically checked, collated, and analysed for abnormal values to form a final analysis database. Correlation analysis was performed using the SPSS 20.0 statistical software package.

All descriptive measurement data are expressed by mean ± standard deviation, and normal distribution data mean comparisons were conducted using variance analysis, and non-normal distribution data mean comparisons were conducted using nonparametric tests (rank-sum tests). The comparisons between the groups were performed using a chi-square test.

## Results

A total of 242 patients were included in this study, including 178 men and 64 women. Their average age was 62.6 ± 8.8 years, and the average number of bypasses was 3.22 ± 0.803. The specific results are shown in Table 1.

**Table 1:** Perioperative baseline data of patients

Items	Sub-item	Percent%	Count	Mean ± standard deviation
Age		100	242	62.6 ± 8.8
Gender	male	73.6	178	
	female	26.4	64	
Bypass count				3.22 ± 0.803
Preoperation Ejection fraction (EF)				62.3 ± 10.3
Left ventricular end diastolic diameter				5.09 ± 0.64
Smoking history		50	121	
Diabetes		40.1	97	
Hypertension		62.8	152	
Chronic renal insufficiency		1.7	4	
Extracorporeal circulation surgery			88	
Cardiac Function Classification (NYHA Classification)				2.44 ± 0.44
Previous percutaneous coronary intervention (PCI)		12.8	31	

This study showed that the proportion of non-sequential single-branched bypass in the right coronary system was the largest (178 cases, 67.9%), followed by the sequential bypasses between the right posterior descending branch and the left ventricle posterior (42 cases, 16.0%). The rest were mostly sequential bypasses between the right crown system and the non-right crown system (including the front descending branch system and the gyroscopic branch system) (see Table 2).

**Table 2:** The right coronary artery system bridg method

	The right crown system bypass method	Frequency	Effective percentage
Group 1	Non-sequential single bypass^a^	178	67.9
Group 2	Non-sequential double bypass^b^	9	3.4
Group 3	Posterior descending branch-posterior branch of left ventricle	42	16.0
Group 4	Sequential bypass with 2 gyro systems and 1 right crown system	1	0.4
Group 5	Sequential bypass with 1 gyro system and 1 right crown system	20	7.6
Group 6	Sequential bypass with one forward descend system and one right crown system	2	0.8
Group 7	Sequential bypass with 1 roundabout system and 2 right crown systems	5	1.9
Group 8	Sequential bypass with 1 forward descending system, 1 gyrating system and 1 right crown system	2	0.8
Group 9	Y-bypass at two target sites in the right crown system	1	0.4
Group 10	Right target system, two target sites, one other system sequential bypass and one non-sequential single branch bypass	2	0.8
In total		262	100.0

^a^In the operation, the right crown system only has a single bypass

^b^In the operation, right coronary system separately set up two bypass

Considering the convenience of subsequent statistics on single-branched bypass and sequential bypass, combined with clinical features, we grouped the intraoperative bypass conditions as follows: the single-nodes of the right-crown system were respectively single-bypassed to the single-group (group A), the sequential bypass of the right coronary system and the convoluted and/or anterior descending branch system were placed in the other systematic sequential groups (group C), while the right coronary system Y-bypass was placed in the PDA-PL right coronary sequential group (group B). The differences in flow, pulsation index and DF parameters between the three groups were compared. Considering that the single crown of the right crown system and one other system have a sequential influence on the flow distribution, this part of the data is ignored (2 cases/0.7%) (see Table 3).

**Table 3:** Intraoperative interventions in the right coronary system

	Group	Frequency	Percentage(%)
Bypass method	Single bypass (Group A)	187	71.9
	Right crown sequential group (Group B)	43	16.5
	Sequential group with other systems (Group C)	30	11.5
	In total	260	100
Right crown bypass material selection	Great saphenous vein	286	92.3
	Radial artery	17	5.5
	Internal mammary artery	6	1.9
	Mix bypass^a^	1	0.3
	In total	310	100
Right coronary target vessel site	Right crown trunk	90	29.1
	Post-fall	147	47.6
	Posterior left ventricular branch	72	23.3
	In total	309	100
Coronary CT bypass occlusion in the week after operation	Single bypass group	11^b^	91.7
	Sequential Group	0	0
	Sequential group with other systems	1	8.3
	In total	12	

^a^The hybrid graft is the posterior branch anastomosis after the right internal mammary artery is connected to the saphenous vein;

^b^Of the 11 cases, 4 were poorly developed, 4 were occluded (grade O), 1 was moderate to severe stenosis (grade B), and 2 were moderately stenotic (grade B)

This study showed that the bypass material used in the right coronary system of our centre was mainly the saphenous vein (286 cases, 92.3%), followed by the radial artery and the internal mammary artery, and the utilization rate of an arterial bypass was low. In the choice of target vessel sites in the right coronary system, we preferred the posterior descending artery for bypass (47.6%), followed by the right coronary trunk (29.1%) and the left ventricle posterior branch (23.3%). One week after the surgery, on the coronary CT examination, 11 cases of bypass vessels had different degrees of poor development or stenosis and occlusion, and this mainly occurred in single bypass vessels (11 cases, 91.7%), of which only one was a sequential bypass blood vessel with other systems. The differences among the above groups were statistically significant (*P* < 0.01) (see Table 3).

In this study, the instantaneous flow of the vasculature in both the right coronary sequential group and the other system sequential group was higher than that of the single group, and the above difference was statistically significant (*P* < 0.01). However, there was no significant difference in instantaneous bypass flow between the two sequential groups (*P* = 0.410). There was no significant difference in the pulsatility index among the three groups (*P* > 0.05). In the diastolic blood supply ratio, the right coronary sequential group was superior to the single-group (*P* < 0.001), and the difference was statistically significant, while there was no significant difference between the right coronary sequential group and the other systematic sequential groups (see Tables 4 and 5).

**Table 4:** Comparison of TTFM parameters of each group in the right coronary system

		Mean	Standard deviation	Standard error	95% confidence interval for the mean		Minimum	Maximum
					Lower limit	Upper limit		
Flow(Q)	Single bypass (Group A)	30.64	20.130	1.472	27.73	33.54	1	110
	Right crown sequential group (Group B)	40.93	20.421	3.114	34.65	47.21	12	127
	Sequential group with other systems (Group C)	45.07	24.458	4.622	35.59	54.56	10	112
	In total	33.92	21.305	1.326	31.31	36.53	1	127
Pulsation index (PI)	Single bypass (Group A)	3.411	3.4503	.2530	2.912	3.910	.8	29.8
	Right crown sequential group (Group B)	3.212	1.6664	.2541	2.699	3.724	1.4	8.1
	Sequential group with other systems (Group C)	4.119	2.2009	.4236	3.248	4.989	1.5	11.1
	In total	3.452	3.1058	.1941	3.070	3.835	.8	29.8
Diastolic blood supply ratio (DF)	Single bypass (Group A)	59.78%	11.8518%	0.8690%	58.065%	61.494%	3.0%	84.0%
	Right crown sequential group (Group B)	66.881%	10.5095%	1.6216%	63.606%	70.156%	38.0%	88.0%
	Sequential group with other systems (Group C)	62.111%	12.1603%	2.3402%	57.301%	66.922%	27.0%	77.0%
	In total	61.196%	11.9237%	0.7467%	59.726%	62.667%	3.0%	88.0%

**Table 5:** Comparison of TTFM parameters of the right crown system (LSD method)

Parameter	Comparison between groups	Mean difference	Standard error	Significance*	95% confidence interval	
					Lower limit	Upper limit
Flow(Q)	A vs B	−10.294*	3.497	0.004	−17.18*	−3.41
	A vs C	−14.435*	4.190	0.001	−22.69*	−6.18
	B vs C	−4.141	5.022	0.410	−14.03	5.75
Pulsation index (PI)	A vs B	0.1997	0.5260	0.705	−0.836	1.236
	A vs C	−0.7072	0.6402	0.270	−1.968	0.554
	B vs C	−0.9069	0.7633	0.236	−2.410	0.596
Diastolic blood supply ratio (DF)	A vs B	−7.1014%*	1.9948%	<0.001	−11.030%*	−3.173%
	A vs C	−2.3315%	2.4048%	0.333	−7.068%	2.404%
	B vs C	4.7698%	2.8803%	0.099	−0.903%	10.442%

*The significance level of the mean difference is 0.05

## Discussion

The development of a CABG surgical strategy is one of the important factors for the success of surgery, of which the selection of target blood vessel sites is the most important strategy. Previous studies have shown that pre-established CABG surgical strategies based on coronary angiography are somewhat different from actual surgical strategies and are not related to the surgeon’s seniority [4]. Regardless of whether the surgeon is a low-grade or high-grade doctor, compared with the left coronary artery system, the actual coincidence rate of the selection strategy of the targeted lesion in the right coronary system is the lowest, and this also shows to some extent that the right coronary revascularization strategy is still controversial. This study found that our centre prefers the posterior descending branch (47.6%). Of course, this may be related to the patient’s coronary lesions, but, on the other hand, may also because that, in off-pump surgery (63.5%), the PDA is more convenient to expose and the intraoperative safety is relatively higher (compared to the right coronary trunk, less arrhythmia induced by bleeding) and other factors.

In recent years, the use of CABG for multiple arterial bypass and whole arterial bypass has become increasingly common, and a large number of studies have confirmed that these patients better mid- and long-term patency rates compared with veins [10, 11]. This study showed that the bypass material for the right coronary system is generally the greater saphenous vein (92.3%), which may be related to its relative safety, relatively simple acquisition, relatively sufficient material, more plasticity, and other factors. The choice of materials for the right crown bridge has always been controversial [12]. Whether the high patency rate of an artery bridge on the left side is also applicable to the right side and the length of internal mammary artery, as well as the acquisition cost and the probability of a high linear sign of radial artery in the near future, all restrict the surgeon’s choice of an artery to a certain extent. Our centre has little tendency to choose multi-arterial bridges, and besides strengthening our understanding, this also needs more clinical practice to shorten the learning curve.

In this study, the flow rate of the comparison between the other system sequential group (group C), the right coronary system sequential group (group B) and the single-group (group A) group gradually decreased, especially in the sequential group and the non-sequential groups, and the differences were statistically significant (*P* < 0.05). To some extent, the viewpoint that the sequential bypass flow was better than the single-bypass flow was verified (as the number of anastomoses increased, the flow rate gradually increased). In addition, in the non-sequential single-bypass, the peak flow of the right coronary target vessel was the highest (37.0 ± 21.6 ml/min), and compared with the posterior descending branch (24.7 ± 15.5. ml/min) and the left ventricle posterior branch (26.9 ± 23.3 ml/min) had a statistically significant difference (*P* < 0.05). However, unlike the previous research results [13–15], this study showed that the left ventricular posterior tributary flow is higher than the posterior descending branch, which is not completely consistent with the classical vascular bed theory. It may be related to the number of cases, causing a certain bias.

Previous studies have shown that due to the larger number of anastomotic ports and relatively larger vascular beds, sequential bypass has a higher flow rate than single-branched bypass, and the high flow rate will delay the proliferation of the intima to a certain extent, making the bypass vessels better with an improved long-term patency rate [13, 15]. One week after surgery, on coronary CT in the study, 11 patients in the non-sequential group had different degrees of stenosis or unclear visualization, but there was only one case in the sequential group. Although not necessarily caused by intimal hyperplasia, this also proves to some extent that high-flow sequential bypasses have better postoperative short-term patency [16].

In this study, coronary CT showed that poor visualization of bridging vessels was more common in radial artery bridges (4 cases). Although the related parameters such as instantaneous bridging flow during the operation were satisfactory, the poor CT results worried the surgeons who intended to carry out multi-artery or whole-artery CABG. On the premise of ensuring the correct strategy, it is still controversial as to which is more important to avoid perioperative linear symptoms by using perioperative antispasmodic drugs and/or a higher perfusion pressure. Whether there are other risk factors affecting the patency rate in the near future still needs further observation and study. This study did not refine and analyse the lesion location or stenosis degree of the right coronary artery, which may affect the surgical strategy and instantaneous blood flow results. In addition, this study is a single-centre study, and the overall sample size is small, so its results need to be further confirmed by large-scale clinical studies.

## Limitations

Our study had several limitations. Due to its retrospective design, we were unable to certify that all potential confounding factors had been recorded. Our single-centre experience may not be applicable to other institutes.

## Conclusion

In our centre, the most common form of CABG in the right coronary artery system is non-sequential SV to PDA. Whether with sequential grafts of PDA-PL or other sequential grafts among the different coronary artery systems, the instantaneous flow of groups of sequential grafts was higher than that of a single graft. DF in the groups of sequential grafts of the right coronary system was better than that in the non-sequential group.

## Data Availability

Data will be made available on request.

## Abbreviations

CABG: Coronary artery bypass grafting
CAD: Coronary atherosclerotic heart disease
PCI: Percutaneous coronary intervention
PI: Pulsatility index
CT: Computed tomography
TTFM: Transient-flow flow meter
MGF: Mean graft flow
DF: Diastolic flow
PDA: Posterior descending artery
RCA: Right main coronary artery
PL: Posterior branch of the left ventricle
LAD: Left anterior descending artery
LCX: Left circumflex
EF: Ejection fraction
